# Suction cup handholds have low efficacy in laboratory evaluation with typical bathing conditions and wall materials

**DOI:** 10.1177/20556683251370322

**Published:** 2025-08-25

**Authors:** Hanaan H. Z. Deen, Iris C. Levine, Roger E. Montgomery, Steven Pong, Alison C. Novak

**Affiliations:** 1 The KITE Research Institute, Toronto Rehabilitation Institute, University Health Network, Toronto, ON, Canada; 2 School of Industrial Design, 6339 Carleton University, Ottawa, ON, Canada; 3 Rehabilitation Sciences Institute, University of Toronto, Toronto, ON, Canada; 4 Department of Occupational Sciences and Occupational Therapy, University of Toronto, Toronto, ON, Canada; 5 Faculty of Kinesiology and Physical Education, University of Toronto, Toronto, ON, Canada

**Keywords:** assistive device, activities of daily living, ageing-in-place, safety, accessibility

## Abstract

**Background:**

Suction cup handholds are sometimes recommended by Occupational Therapists for bathing transfers when permanent grab bars are not feasible. The efficacy of suction cup handholds on typical bathroom surfaces is unknown.

**Purpose:**

This study evaluated the efficacy and failure characteristics of two brands of suction cup handholds on six typical bathtub wall surfaces, under environmental and loading conditions associated with bathing.

**Method:**

Eighteen suction cup handholds underwent controlled longevity testing under wet and dry conditions in separate sessions. Handhold efficacy was evaluated through (a) visual inspection, (b) manual manipulation, and (c) controlled loading, with days to failure and other failure details as outcome measures.

**Findings:**

No handhold-wall sample combinations were effective over the 28 day test period, with at least one handhold on each wall sample failing on day one. Handhold failure was most frequently due to sliding along the wall surface, and occurred most frequently during manual manipulation testing.

**Conclusion:**

Handhold efficacy was poor in a controlled experimental environment. These results can inform clinicians of the risks suction cup handholds pose and assist in clinical recommendations against their use.

## Introduction

Falls are a serious public health problem, being the second leading cause of unintentional injury deaths worldwide.^1^ In 2022, the Public Health Agency of Canada reported 78 076 fall-related hospitalizations among adults aged 65 years and older, representing 88.6% of all injury-related hospitalizations among this age group.^2^ Aminzadeh et al. found that 15% of seniors in their study experienced falls within the bathroom while Stevens et al. found that of falls within the bathroom, 70% of those occur within the bathtub or shower.^3,4^ Environmental factors affecting risk of fall-related injury in the bathroom include slippery floors, hard surfaces and sharp edges on features such as bathtubs, and inadequate or absent aids such as grab bars.^5,6^

Grab bar installation in the bathing environment is recommended for support to counter the potential loss of balance during bathing transfers, allowing for independence.^7^ Grab bars are commercially available in different forms, such as permanent (i.e. bolted to the wall) and temporary grab bars (i.e. suction cup handholds). Permanent grab bars have regulations regarding installation as stated within Accessibility Standards Canada^8^ which ensures a stable base of support during transfers. However, such standards along with factors such as cost, accommodation restrictions (i.e. rental rules regarding permanent changes), installation challenges (i.e. technical skill requirements) and/or social pressures provide a barrier to installation^3,9–12^ which can lead users to seek temporary alternatives such as suction cup handholds. Suction cup handholds seem convenient for users due to their easy installation, and Guay and colleagues^13^ have measured forces applied to grab bars in an experimental setting to average 23.2% body weight, and suggested that the loads observed does not exceed the ISO loading requirements specified for suction cup handholds.^14^

However, some clinicians have highlighted suction cup handholds as a potentially hazardous tool and are reluctant to recommend them for transfers as they have observed in clinical practice that their sturdiness is questionable and they fall off the wall or do not perform as expected.^12^ Along with the observed functional limitations, Rand et al. found that older adults (i.e. the target population of the suction cup handhold product) may require additional guidance on where and how to install suction cup handholds effectively to support bathing transfers.^15^ While some older adults may have assistance from others to install and maintain suction cup handholds, it is important the target population of such products are able to install and maintain them their selves. However, despite these observations, suction cup handhold products are still marketed as effective for supporting bathing activities. It is unclear whether ineffectiveness of suction cup handholds observed in these studies is grounded in the performance of the suction cup handhold, or whether other factors (e.g. inappropriate installation) contribute to perceived poor performance. The efficacy of suction cup handholds when used under standard bathing conditions is being studied to support clinicians in the prescription of bathing aids.

Mechanically, suction cup performance is sensitive to a number of environmental factors, which are challenged under typical bathing conditions. The suction cup handholds often advertised for use within bathrooms are passive suction cups which rely on an external pushing force for the suction cup to adhere to a surface. To detach the suction cup, the external pulling force has to exceed the maximum air pressure force between the suction cup and the surface.^16^ Therefore, the load a suction cup can sustain is dependent on suction cup adhesion, which is sensitive to humidity, water exposure, and temperature. During bathing, the relative humidity of the bathroom can exceed 100% and temperatures can rise to 30°C.^17^ Increasing humidity can increase the adhesion force of the suction cup^18^ until relative humidity reaches approximately 70%, beyond which the adhesion force is observed to decrease.^19^ In contrast, submersion under water may improve suction cup adhesion,^20^ although full submersion of a suction cup handhold is not typical during bathing. Classically, temperature affects the flexibility of the materials used to construct suction cups with lower temperatures leading to a stiffer material.^21,22^ Suction cup stiffness is a tradeoff, with the suction cup needing to be compliant enough to conform to the surface and form a seal, without it being too compliant which is seen to limit the cups ability to resist deformation leading to failure.^23^

As indicated in the manufacturer documentation, suction cups are also sensitive to the material, texture or cleanliness of the surface they are adhered to as these variables impact the available contact area and consequently the suction cups adhesion.^23,24^ Many temporary handholds include instructions for use on smooth, non-porous surfaces, and within areas larger than the suction cup pad to avoid installation over grout lines and beveled edges. A scan of home improvement websites indicate that common bathroom wall materials include acrylic, ceramic, natural stone, glass, porcelain and metal in varying sizes and finishes, which may not match the recommended surface requirements for suction cup adhesion. Porous material, materials with surface texture and roughness, and tiles smaller than the suction cup pad or mosaic style tiles would result in poorer adhesion due to air passage between the suction cup and surface.^25^

Considering all factors affecting suction cup adhesion and consequently the load they can support, it is unknown if suction cup handholds would be effective bathing aids. Humidity, temperature, and water may increase or decrease adhesion, while the wall materials available for consumers to use within their bathrooms may not be ideal for suction cup use. The physical limitations, along with skepticism from clinicians due to observed failures,^12^ and missing manufacturing or installation regulations supports a knowledge gap that must be filled to allow clinicians to be confident in their clinical recommendations.

To date no study has reported the efficacy of these devices under loading and environmental conditions associated with bathing. This study aimed to determine the performance of two suction cup handholds on six typical bathtub wall surfaces under environmental and loading conditions associated with bathing. A secondary goal was to describe the failure characteristics of the handholds.

## Method

This experiment took place in CareLab at the KITE Research Institute at the University Health Network. CareLab is a simulated hospital single patient room with a fully functional bathroom. The bathroom in CareLab consists of a 1.37 m × 0.9 m × 1.6 m alcove shower where a custom designed rectangular prism framed rig was placed within and used to mount the sample bathtub wall surfaces.

**Figure 1.**
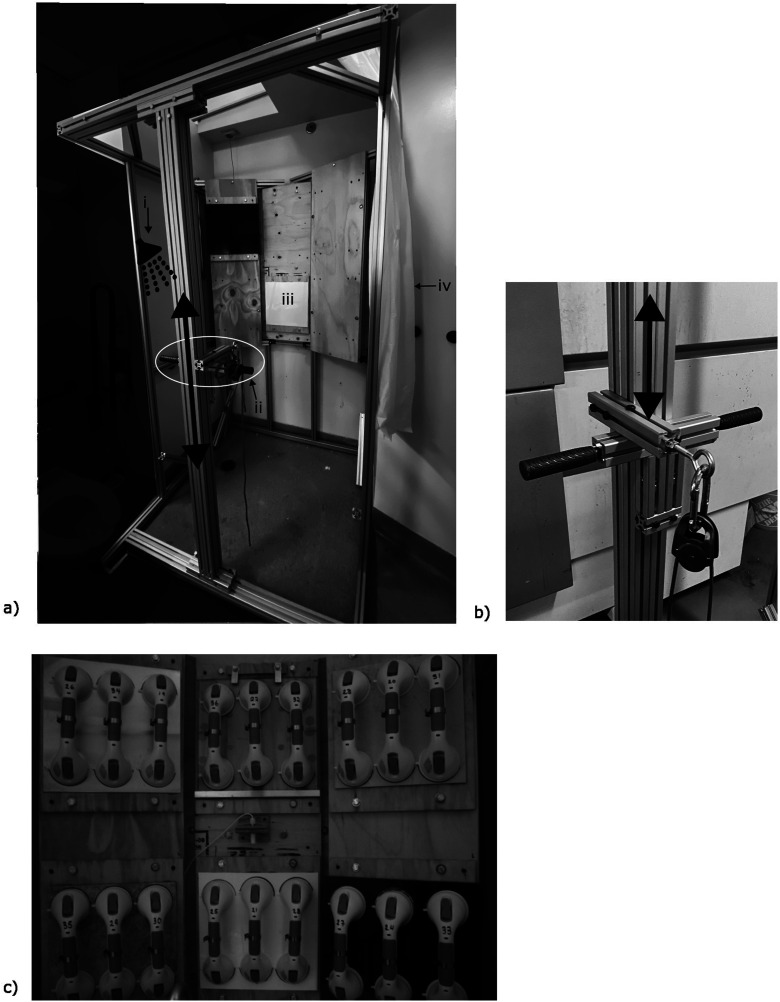
Rig frame inside alcove shower. (a) Rig frame inside alcove shower with labels corresponding to the following elements of the rig and bathroom setup: (i) Shower head relocated to allow even distribution of steam around all handholds, (ii) the white circle encompasses the pulley system and height adjustment track, with arrow indicating that the pulley is able to adjust vertically within the track, (iii) bathroom wall surface samples adjacent to shower controls, (iv) shower curtain that would be draped around entire rig during testing. (b) Close up image of simple pulley system. Handles allow system to slide up and down within gaps of aluminum extrusion. (c) Six wall samples affixed to interchangeable plywood backings. Three suction cup handholds randomly selected for each of six wall samples. The panels on the right and left side tilted 45° along vertical axis to allow a consistent angle of attachment for the pulley system. The temperature and humidity sensor is visible in the centre of the set up. Hose clamps are seen affixed to the middle of each handhold for consistent point of attachment.

The back wall of the rig housed the bathtub surface samples and was positioned on the wall perpendicular to where the shower controls and shower head were located (Figure 1(a)). The back wall of the rig was fitted with six interchangeable plates housing the wall surface samples, organized into two rows containing three plates each. The wall surface samples were mounted to a plywood backing with construction adhesive, which in turn was mounted to the rig with bolts. The bolts allowed the position of each sample to be selected randomly and changed between testing conditions to limit the effect of sample position on the outcomes of the study.

To further prevent the effect of sample position on the study outcome, the shower head was repositioned to be equidistant from all wall samples to allow steam to be generated evenly around each. To control the amount of water exposure to each handhold through protocol condition (described below), handholds were positioned to avoid any water splashing directly on them. As the water and steam for the experiment was generated from a functional shower, the temperature and humidity could not be precisely controlled. A UBIBOT temperature and humidity monitoring system (UbiBot ® WS1 Pro, Dalian Cloud Force Technologies Co Ltd., Dailan, China) was used to monitor the conditions each day for consistency. Shower curtains were draped around the rig to retain consistent temperature, humidity, and steam in the environment.

Opposite the wall of surface samples was a simple pulley system which applied a force of 126.7 N at a downwards angle of 33° from the horizontal to the handhold on each wall sample to simulate the average entry and exit force applied to vertical grab bars during bathtub exit. These values are consistent with the loading observed by Greene and colleagues^25^ during an unperturbed bathtub exit using a vertical grab bar (8.9% body weight), scaled to a 95th percentile male (1263.5 N).^26^ The vertical position of the pulley system was adjustable to allow the same downwards loading angle for the top and bottom rows of samples (Figure 1(a), 1(b)). Wall samples were arranged horizontally into two rows of three, with the 1^st^ and 3^rd^ wall sample in each row placed at a 45° angle to ensure the load was applied perpendicular to the plane of the tile, allowing for the same load application in the same orientation for each handhold. Hose clips were attached to the middle of each handhold to ensure a consistent point of load application (Figure 1(c)).

### Materials

**Figure 2.**
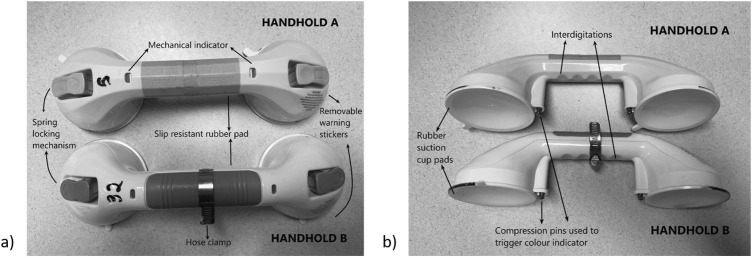
Visible (a) and reverse (b) sides of Handhold A and B with labels of the similar features.

Two different brands of commercially available suction cup handholds were used in the experiment. Eighteen copies of each brand of suction cup handhold were evaluated (i.e. 36 handholds, total). Handhold A and B measured 0.3 m × 0.11 m (12″ × 4 ¼″) and 0.31 m × 0.10 m (12.2″ × 4″), respectively, with each suction cup pad for both handholds measuring 0.092 m (3 ⅝″) in diameter. Handhold A weighed 0.34 kg and Handhold B weighed 0.35 kg. Both handholds featured many similar components (Figure 2(a) and (b)), including dual-locking suction cups, mechanical indicators which change from red to green when a spring is sufficiently compressed, slip resistant rubber pads, plastic interdigitations, and removable stickers with identically-worded warnings regarding the use of the handhold for balance only. The two rubber suction cup discs of each handhold were attached to springs, each controlled by a plastic lever. To install the handhold, the user is directed to clean the rubber discs and wall surface, then press the discs to the surface and depress the levers and check if the device is secure before use. The devices differed minimally in length of graspable area and cosmetically in appearance. Models of Handhold A were assigned identifiers 1–18, and Handhold B were assigned identifiers 19–36.

For both suction cup handholds, the instructions provided by the manufacturer specified that the handhold must be applied to an (a) smooth, (b) non-porous surface, (c) non-textured surface, with (d) at least 1.3 cm or ½” gap between the edge of the suction pad and any gap between tiles. Following these requirements, commonly purchased bathtub wall materials were identified by filtering through online home improvement retailer catalogs by material and sorting by most popular items. To filter the material down to six options, the team used preliminary testing to identify if the suction cup handhold would attach onto a material before selecting it for the trial. The materials that were excluded from further testing included vinyl, lightly textured ceramic (not marked as textured in advertising), laminate, drywall, and painted drywall, as the handholds did not adhere. The final six wall samples that were used in testing represented commonly available shower materials, and included clear glass, smooth ceramic, smooth porcelain, acrylic shower surround, sealed natural stone (granite) and stainless steel (Table 1).

**Table 1.** Material properties of wall samples used in testing, as described by manufacturers.

			Properties	
Sample	Texture	Finish	Dimensions (length x width x thickness)	Edge type
Clear glass	Flat glass, smooth	Polished	0.36 m × 0.36 m × 0.01 m	Flat
Smooth ceramic	Smooth	Glazed	0.34 m × 0.34 m	Pressed
Smooth porcelain	Non-textured	Glazed	0.33 m × 0.33 m	Non rectified
Acrylic shower surround	Smooth	Sealed	0.3 m × 0.3 m × 0.003 m	Flat
Sealed natural stone (granite)	Smooth	Polished	0.41 m × 0.41 m	Rectified
Stainless steel	Non-textured	Brushed finish	0.36 m × 0.36 m × 0.002 m	Flat

### Protocol

Testing was conducted in four sessions, two sessions for each brand of handhold. For each handhold, one session involved pouring 0.15 L of water over the suction cup pads to simulate potential splashing or dripping the handhold would experience from typical use if it were placed in an area closer to the water source, or if the user did not dry their hands before use. The other session simulated the handhold experiencing no direct water poured onto it, as in the case when the handhold is placed further from the water source and hands are dried before use. The planned duration of each session was up to 28 days, as it was anticipated that a typical user might remove and replace the handhold as part of typical bathroom cleaning (i.e. at least once per month). Testing for a handhold was determined complete when the handhold failed (see below for details)

Three handholds of the same model were randomly assigned to and placed on one of six wall surface samples. Samples and handholds were both cleaned with a commercially available unscented bathroom cleaner (Scrubbing Bubbles Free Bathroom Cleaner, S.C. Johnson & Son, Racine, USA) using a non-abrasive sponge. After scrubbing the handholds and wall surface samples, they were rinsed and left to air dry, then inspected for imperfections (scratches, broken seals, cracked levers). The handholds were then placed on the wall surface samples with the suction pads outlined with a waterproof marker to ensure a consistent orientation and identification throughout the study. Metal hose clamps were placed at the midpoint of the graspable portion of the handhold to control the attachment point of the loading mechanism.

During individual testing days, a multi-stage simulated shower and failure testing protocol was carried out with both failure means (i.e. how the handhold failed) and failure timepoint (i.e. during which test the handhold failed) recorded independently for each handhold. The procedure occurred at approximately the same time each day, within a standard deviation of 1 h 9 min across all sessions. At the beginning of each session, a photo was taken of all the suction cup handholds, and the initial temperature and humidity were recorded. Then the suction cup handholds were visually inspected to determine if the device’s suction had released between testing session or if the suction pad had moved between testing relative to the marker outline.

After the initial inspection determining if there was failure between testing sessions, the shower knob was turned to a marked position. The shower was kept on for a total of 13 min: the first 3 min used to allow the shower to reach a comfortable bathing temperature and the additional 10 min to simulate a shower of this length. Peak temperature and peak humidity were collected, independently, during the 10-min shower period.

Following the simulated shower, the researcher manually manipulated the handhold by applying a low oscillating force up, down, left and right. This step was specified in the suction cup handhold user manuals to help ensure the handhold is secured to the surface. This step also posed as an extra safety process for the research team to reduce the risk of sudden loss of suction during the loading phase of the trial. If movement of the handhold was noted by the researcher during this manual manipulation, the handhold was deemed to fail. If failure was not identified following manual manipulation, a load simulating typical bathing use was then applied using the pulley system. The carabineer at one end of the rope connected to the load was attached to a rope looped around the hose clip on the middle of the handhold. The load was carefully lowered to reduce the amount of jerk applied to the handhold and was left static for 5 seconds, then carefully removed. The handholds on each wall surface sample were load tested, with the order of wall surface samples randomized each day.

After loading tests were complete, a photo was taken in the same orientation as the photo taken at the beginning of the trial. The condition of each suction cup handhold, failed or not failed, was recorded at three points each trial day: after the visual inspection, after the manual manipulation test, and after load application. Handhold failure means was categorized as follows: *Fell off*, where the handhold fell off the wall surface sample completely where both suction cups failed, with no external loading applied. *Indicator failure*, where the visual indicator changed from green to red without there being any visible suction loss or suction cup movement. *Sliding*, if the handhold slid, with one or more suction pad moving outside of the marked outline while retaining suction. *Suction loss*, where one suction cup failed as determined by there being a gap between the suction pad and the wall during external loading, with or without indicator change. Failure means could have occurred during any point each trial therefore both failure means and failure timepoint were recorded for each handhold upon failure.

### Data analysis

The study was designed to allow a three-way repeated measures ANOVA, with wall surface sample and suction cup handhold as between-subjects factors and liquid condition as a repeated measure, and number of days as the dependent variable. ANOVA was also used to evaluate temperature and humidity between timepoints (starting and peak) and sessions. Descriptive statistics (means, standard deviations and counts) were used to summarize outcomes. All statistics were analyzed in MATLAB R2023a Version 9.14, with α = 0.05.

## Results

No handhold-wall surface sample combination reached the plan session duration of 28 days, with the best performing handhold-wall surface sample combination lasting 8 days. Based on this outcome, the planned ANOVA analysis was not completed for handhold longevity, as the performance of all products was poor, and therefore statistical differences would have little clinical importance. The temperature and humidity conditions of the testing sessions are summarized in Table 2.

**Table 2.** Mean (SD) of temperature and humidity recorded each session.

	Session 1	Session 2	Session 3	Session 4
Handhold	A	A	B	B
Water application	Yes	No	Yes	No
Starting temperature (°C)	24.0 (0.5)	24.2 (0.3)	23.9 (0.4)	24.4 (0.3)
Starting humidity (RH)	46.8 (8.1)	37.0 (4.3)	58.7 (9.5)	37.7 (8.4)
Peak temperature (°C)	25.5 (0.5)	25.6 (0.2)	25.3 (0.8)	25.5 (0.5)
Peak humidity (RH)	78.4 (4.5)	78.4 (4.3)	71.2 (6.0)	71.3 (3.5)
Total session length (days)	8	6	4	5

There was a significant effect of timepoint (starting vs peak) on temperature (F = 690.5, *p* < 0.001), such that peak temperature was a mean of 6.1% (SD 1.2%) higher than starting temperature, across days and sessions. For humidity, there was a significant disordinal interaction of day by timepoint, timepoint by session, and day by timepoint by session. Upon further review, this appears to be driven by substantial day-to-day variation in humidity (starting coefficient of variation, 24.4%; peak coefficient of variation, 7.3%) within the building due to possible factors such as weather conditions, air conditioning, and increased air filtration for infectious diseases. When interactions were broken down by combinations of day and session, humidity differed significantly between timepoints (all *p* < 0.05), However, more succinctly, there was an overall significant effect of timepoint on humidity (F = 696.5, *p* < 0.001), with an average humidity increase of 77.7 % (SD 39.4%; or average (SD) increase of 30.8 (10.8) percentage points) between start and peak humidity, across handholds and sessions. There were no main effects of day or session for temperature or humidity (all *p* > 0.05).

**Figure 3.**
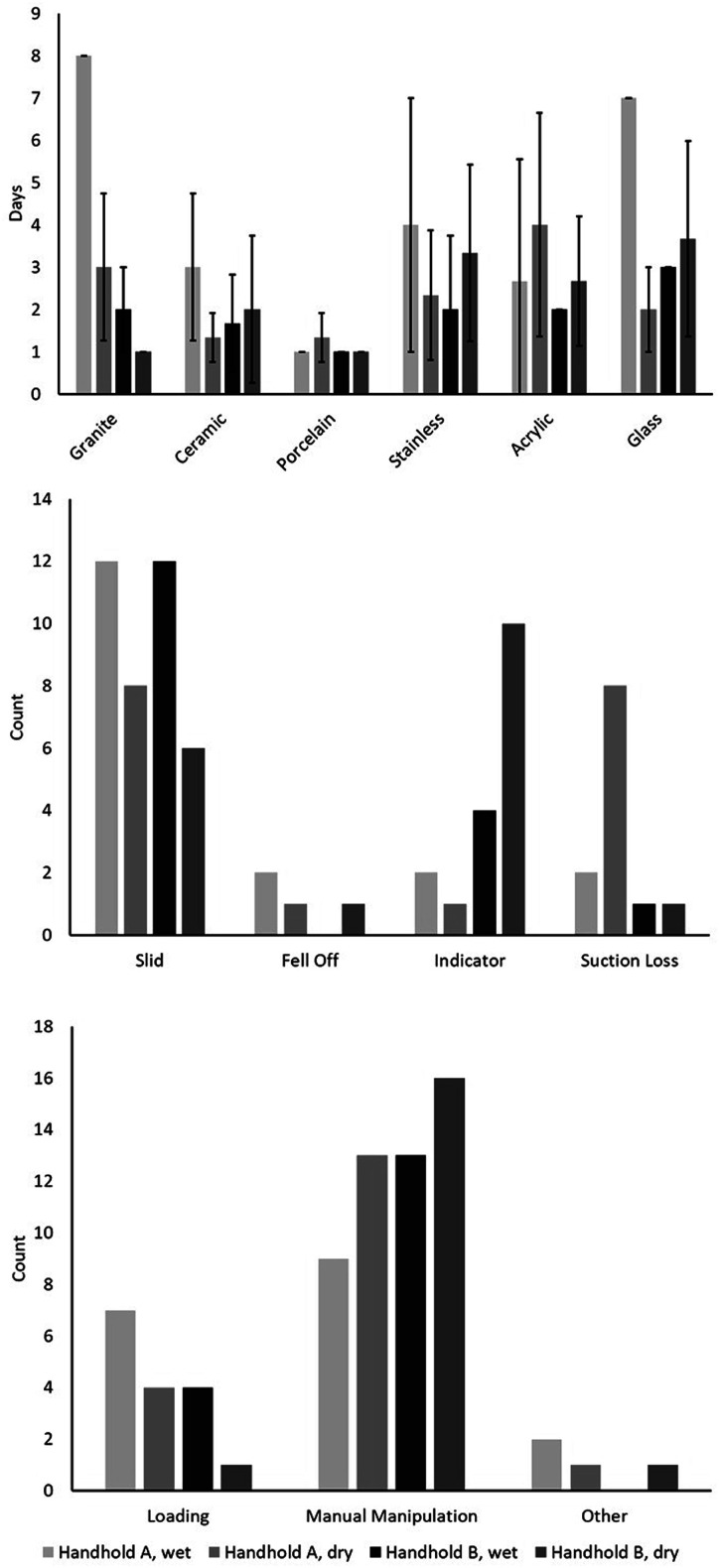
(a) Days to failure for handholds on each wall sample. Whiskers indicate standard deviation for days to failure. Frequency of (b) failure means and (c) failure timepoints over sessions (grey bars).

Each session ranged a maximum of 4–8 days before all the handholds failed (Figure 3(a)). The longest session, Session 1, lasted 8 days and had 10 handholds (56%) fail by the end of day 4. Sessions two and three had more than half of the handholds (67%) fail by the end of day 2 and Session 4 had more than half of the handholds (61%) fail by the end of day 1. The days to failure for each wall surface material were inconsistent between sessions. As an example, the handholds on the granite sample were failing anywhere between 1 and 8 days and the handholds on the glass sample were failing between 1 and 7 days. The other samples had similarly large ranges for days till failure with only the porcelain sample performing similarly between all sessions, having 11 out of 12 handholds fail on day 1.

More than two thirds (67%) of the failure means for the wet conditions for both types of handholds (Sessions 1 and 3) were attributed to sliding (Figure 3(b)). Other failure means were indicator failure (24%), suction loss (17%) and falling off (6%), with the percentages representing data from all collection sessions. More than half of the handholds (72%) failure timepoints were during the manual manipulation test with the loading test resulting in 22% of failure timepoints (Figure 3(c)). The remaining 6% of handhold failure timepoints were at moments when a direct force was not applied to the handhold including: visual inspection at the beginning of the trial, while the shower was running, or between testing sessions. These failure timepoints outside of the manual manipulation or loading tests were labelled as *Other* in Figure 3(c).

## Discussion

This study aimed to evaluate the efficacy of two suction cup handholds on common bathtub wall surfaces under realistic temperature, humidity and wetness conditions. During testing all handhold-wall surface sample combinations had at minimum, one of three handholds fail on the first day of testing. Due to the overall poor performance of all suction cup handholds, insufficient data was gathered for analysis of longitudinal differences between handholds, conditions or wall samples. As a result, the performance of the handholds under real-world conditions remains unpredictable; however, the descriptive statistical outcomes which show large rates of handhold failure within the first few days indicate suction cup handholds are not appropriate for use within the bathing environment.

The timepoints when handholds failed most frequently was during manual manipulation, the first step of each testing session. The user manuals included with the suction cup handholds indicated that the devices should be tested using manual manipulation prior to use, meaning handholds would not be immediately usable upon a cycle of cleaning and installation if the handhold were to fail the manual manipulation test through any means (i.e. sliding, suction loss, indicator failure, other); the handhold would only be usable after a successful repetition of the cleaning, installation and manual manipulation steps. Failing this, the handholds should not be used for balance recovery or support during bathing tasks and transfer, both being main purposes of grab bars.^27^ The handhold failing during manual manipulation and requiring repeated re-installation until sufficient adhesion is reached can become fatiguing to users or may not be possible for users who relied on external support to install and maintain the device. If a successful installation is not reached, it could result in the use of an improperly installed aid which could fail during transfers leading to injury or leave users without an aid during bathing which could increase the risk of falls.

Furthermore, we observed several means of handhold failure, which have differing implications for use. More than half (53%) of failures occurred due to *sliding* of the suction pads across the wall sample, a failure mean which may be more challenging to observe in a realistic environment without controls such as the marker outline or daily photo documentation. Though the sliding distance was not quantified, any indication of sliding should be deemed as failure as the adhesion force is decreased which result in lower load bearing capabilities.^16^ A concern of this failure means is that users may reach for the handhold during bathing tasks, or for use in balance recovery, and it may not be in its expected position. Higher handrails may provide a stability advantage,^28^ and most grab bar users prefer to install^15^ and grasp^29^ grab bars at approximately shoulder height for bathing transfers. With handholds sliding down bathroom walls, users may be expected to use a higher physical demand for balance recovery as seen with handrails of lower height. Given the importance of stored visual information for reactive grasping,^30^ it is critical for an assistive device to remain in the anticipated location. The handhold sliding during use is also a problem as users are expecting a stable surface to support them during their task. If the handhold were to slide around while in use, it may lead to a loss of balance for the user. Grab bar position affects balance control through modification of the moments of force and center of pressure.^31^ Sliding of a grab bar may require instantaneous compensatory adjustments, which could exceed a user’s strength or power.

Handhold failure due to *suction loss* during the mechanical loading test indicates that the handholds are not able to support typical bathing entry and exit transfers. The handholds we tested did not specify a loading limit, but did specify they are unable to support full body weight. Users may logically assume that during a transfer, they will not be applying their entire body weight and attempt to use the device when in fact, it cannot consistently support the typical loading force applied to a vertical grab bar during entry and exit transfers (8.9% body weight)^25^ for the 95th percentile male. The handhold failing during bathtub transfers can cause a harmful fall onto hard bathroom tiles or sharp corners.^5,6^ The manual suction cup handholds design is likely unable to produce a sufficient adhesion for secure transfers as it lacks an active suction mechanism. Suction cups used in industrial applications for heavy loads are able to do so through multiple methods such as vacuum pumps within the system.^32^ To improve suction cup handhold design, an active suction element can be added to produce a higher adhesion force which would allow for the handhold to sustain higher applied forces.

*Indicator failure* was another reason handholds were deemed to have failed testing. The indicator colour patch on the handhold is used to tell the user if the suction cups are securely attached to the surface and safe for use, however, the mechanism relies on compression of a spring behind the suction cup pads and compression pins which depress in relation to the suction cup pads. In 25% of cases, the spring decompressed when suction was not adequate, and the indicator changed from green to red, effectively indicating that the suction was not secure. However, in the balance of cases (75%), the spring remained compressed even though suction was lost, and the indicator did not reveal that suction had not been maintained, and is therefore not a reliable safety indicator. In a study conducted by Rand and colleagues,^15^ older adults were asked to install similar suction cup handholds. Some participants expressed that the indicator was too small to see clearly, or was challenging to interpret and were unable to determine based on the indicator whether the handhold was installed correctly. A failure indicator is a helpful feature; however, the implementation of this feature must be redesigned to accurately and clearly indicate suction loss.

We observed several design elements of the handholds that affected performance and may further suggest they are not appropriate bathroom aids. The lever mechanism of the suction cup head of both handholds allowed water to enter within the suction mechanism, which may cause damage to internal spring components and lead to other elements, such as the suction indicator, to become faulty. In addition, the main element of the suction cup handhold, the rubber suction cup pads, are recommended to be stored at temperatures between 15°C and 25°C with humidity conditions below 65% and for both very moist and very dry conditions to be avoided as stated in the International Organization for Standardization 2230 Rubber Product Guidelines for Storage.^33^ The temperature during the experiment was within the upper range in which rubber is meant to be used; however, the humidity by the end of the trial was higher than the maximum recommended value. Specifications regarding safe temperature and humidity operating ranges would be valuable for the user to determine whether the product is appropriate for their bathroom, as use in suboptimal condition may result in weaker suction cup performance.

The short time to failure of the handholds and the inability to identify factors which could allow for effective handhold use should be reviewed by clinicians when considering a suction cup handhold as a bathing aid. Permanent grab bars are more stable as they can be securely installed to a stud or solid backing^8^ and are shown to reduce fall risks in laboratory studies.^34^ The limitations of their counterpart, temporary grab bars and in this study suction cup handholds, were seen when used within the laboratory setting. The handholds failed in multiple ways which may increase the risk for falls. Handhold sliding may result in failed balance recovery as the user can reach for an aid in a position it has moved from whereas the concern of handhold failure during loading is clear as users cannot trust that the handhold will remain adhered during bathing transfers and balance recovery. Failure of an assistive devices during these tasks can increase the challenge of these already difficult tasks and may lead to more serious injuries which are commonly seen in the bathroom due to the hard surfaces and sharp corners.^5,6^ If clinicians want suction cup handholds to be a viable option, the products must be redesigned for balance recovery and with the loading capacity to sustain bathing transfers^25^ as well as designed for use within the environmental conditions found within the typical bathroom.^17^

### Limitations

Our study of the efficacy of suction cup handholds on common bathroom wall surfaces has several limitations. The overall performance of the handhold was poorer than expected with handholds failing quickly which prevented the comparison of days to failure between materials, handholds and water conditions. This prevented us from being able to understand factors which affected performance. Furthermore, the testing protocol included a limited number of wall and handhold samples and was tested under only two environmental conditions. The wall samples selected may not represent the full diversity of products in Canadian homes. We eliminated many wall surface samples prior to testing due to surface characteristics while others were not available in dimensions which met sizing requirements. These products would also not work in a real-world setting, as the suction cup pads could not be placed a sufficient distance from tile or grout edges to ensure a secure suctioning area. While the stainless steel we tested is less common in typical home settings, it is more commonly used in commercial and institutional settings,^35^ and in mobile homes^36^ whose numbers totaled 150,000 in Canada in 2001.^37^ Moreover, the limitation in handhold models tested stems from the restricted space available on each wall sample to test handholds of larger size, both in overall length and suction cup width. However, given that suction cup handholds with longer handles between suction pads would have the potential to generate larger moments of force at the suction pads, it could be inferred that the shorter models we tested would perform better than longer models. Conversely, while models with more suction pads or a shorter handle distance may improve loading performance, they would limit the area available for hand placement, which may reduce effectiveness as a balance support in real-world scenarios.

A further limitation of the handholds is that the age of the device is unknown. Based on ISO 2230 and its application in industry, natural rubber has a shelf life of 5 years^33^ while rubber suction cups are recommended by one manufacturer to be used within 24 months.^38^ It was unclear when the handholds had been manufactured, or if the age of the handholds affected their performance. There were no indications on any of the handhold packages, documentation, or the handholds themselves that could be used by an end user to determine whether the handholds are new enough to be effective.

Equally important, the limitation of testing only two environmental conditions (wet vs. dry) and being unable to control for temperature and humidity prevented an understanding of suction cup efficacy if used under environmental conditions which are more optimal for maintaining adhesion such as bathing with humidity under 65%–70% relative humidity and maintaining temperatures between 15°C and 25°C.^19,33^ However, it is unrealistic to assume these ideal environmental characteristics would be monitored or maintained in a real bathroom. In situations of better adhesion, the user must inspect the handhold prior to each use as instructed by manufacturers, and reposition it to maintain strong suction whenever the handhold fails; however, we did not test whether frequent repositioning of the suction cup handholds would cause wear to components, or change the overall performance of the suction cup handhold.

Additionally, the testing protocol included the manual manipulation test as indicated in the handhold instruction manuals as a preventative measure to reduce the risk of suction cup failure during loading. While we maintained the same researcher to perform manual testing and ensure consistency, the person responsible for completing the test (female, age 35 years) may not be representative of the population who might use a suction cup handhold. Users with a lower grip or upper body strength, poor balance, or using a more tenuous approach may apply lower loads during manual manipulation, which may increase the probability of handhold failure during a bathing transfer rather than during manual manipulation.

Lastly, we were unable to evaluate the micro-damage to the study material throughout the trials. We observed a decrease in session duration from Session 1 (8 days) to Session 4 which may have occurred due to unobserved microscale changes to the handholds or wall surface samples, such as scratches or removal of finishes. Evaluating such micro-damages could reveal potential implications of the use of suction cup handholds with older tiles which may be scratched or stripped of finishes.

## Conclusion

This study was the first to investigate the efficacy of suction cup handholds in regards to loading capabilities, longevity, and failure means when in use in a simulated bathing environment under typical bathing conditions. The observations in this study indicate suction cup handholds may not be effective for bathing transfers. The handholds evaluated did not consistently stay attached to the bathroom wall surface samples for more than 1 day, and slid or fell off under conditions of typical use. Additional design features, such as indicator mechanisms, were not consistently effective. The overall effectiveness of suction cup handholds for use in typical bathing environments and conditions may be limited. Clinicians considering suction cup handholds should evaluate the appropriateness of the products relative to more permanent solutions, when possible, and should consider messaging to clients regarding installation, manual testing of suction cup handholds, and possibility of failure resulting in a fall, in order to ensure clients are informed about the potential risks of suction cup handholds.

## Key Messages

• Typical bathing conditions are outside the ideal ranges for use of suction cup devices and can lead to device failure.
• Suction cup handholds are likely unable to consistently support users during bathing tasks and are not suitable for multi-day use.
• An inaccurate suction indicator poses risks to the end user.
